# Expansive Soil Crack Depth under Cumulative Damage

**DOI:** 10.1155/2014/498437

**Published:** 2014-03-04

**Authors:** Bei-xiao Shi, Sheng-shui Chen, Hua-qiang Han, Cheng-feng Zheng

**Affiliations:** ^1^State Key Laboratory of Simulation and Regulation of Water Cycle in River Basin, China Institute of Water Resources and Hydropower Research, Beijing 100038, China; ^2^Geotechnical Engineering Department, Nanjing Hydraulic Research Institute, Nanjing 210024, China; ^3^College of Resource, Hebei University of Engineering, Hebei University, Handan 056038, China

## Abstract

The crack developing depth is a key problem to slope stability of the expansive soil and its project governance and the crack appears under the roles of dry-wet cycle and gradually develops. It is believed from the analysis that, because of its own cohesion, the expansive soil will have a certain amount of deformation under pulling stress but without cracks. The soil body will crack only when the deformation exceeds the ultimate tensile strain that causes cracks. And it is also believed that, due to the combined effect of various environmental factors, particularly changes of the internal water content, the inherent basic physical properties of expansive soil are weakened, and irreversible cumulative damages are eventually formed, resulting in the development of expansive soil cracks in depth. Starting from the perspective of volumetric strain that is caused by water loss, considering the influences of water loss rate and dry-wet cycle on crack developing depth, the crack developing depth calculation model which considers the water loss rate and the cumulative damages is established. Both the proposal of water loss rate and the application of cumulative damage theory to the expansive soil crack development problems try to avoid difficulties in matrix suction measurement, which will surely play a good role in promoting and improving the research of unsaturated expansive soil.

## 1. Introduction

Expansive soil is a special unsaturated soil which is fractured and has significant swell-shrink characteristics and over-consolidation, and it situates in the slope area of expansive soil, including the embankments, the cuttings, and the channel slopes, it often leads to landslides, bringing huge disaster to project construction. After systematic research [1–5], scholars think that under atmospheric forces, the wet-dry cycle makes the expansive soil swell in wet and shrink in dry, the soil body cracks when the tensile stress exceeds its own resistance to tension, which weakens the soil structure and provides favorable conditions for rain infiltration. As a result, the soil's shear strength and the slope's safety factor drop significantly, the project properties deteriorates significantly and causing frequent occurrence of expansive soil project disasters.

Cracks can control the expansive soil strength, the final crack depth, which is key to expansive soil's project disaster prediction and prevention, is closely related to the expansive soil's slope instability slide depth. For the law of crack formation, to carry out research of the crack depth and its morphological description has also gradually becomes a key elements in expansive soil slope stability analysis. Yin et al. [2, 3] analyzed the affecting factors of crack formation and discussed quantitative methods of cracks, thinking that the generation and development of crack was a key factor for expansive soil slope damage. Starting from morphology, Tang et al. [4–6], analyzed the crack development law and predicted the location where crack may be formed. Zhang et al. [7–9], analyzed the crack evolution laws, and results showed that the key factor that affected cracking depth and stretch degree was not the soil moisture, but its change rate. The final crack depth is closely related to the expansive soil's slope slide damage degree; Lee et al. (1988) used the approach of linear elastic fracture mechanics to calculate ideal soil crack length, proving the applicability of linear elastic fracture mechanics in studying soil body cracking. Morris (1992) [10] tried to use approaches like the linear elastic mechanics, the linear elastic fracture mechanics, and shear failure to study soil cracking length. Yao et al. [11] gave a preliminary answer to clay cracking in elasticity and linear elastic fracture mechanics. Zheng et al. [12] studied the cracking mechanism and the initial cracking depth quantitative expression of expansive soil under the influence of climate.

The crack development process is analyzed in all the above-mentioned researches, and the affecting factors of crack formation are also studied. Meanwhile the moisture content changes is considered to cause matrix suction increase inside the soil body, when the expansive soil shrinks due to water loss, crack will appear because the tensile stress increases to exceed the tensile strength of the soil itself, but the time factor of water content change is not considered. In addition, it is also pointed out in the above-mentioned researches that the initial expansive soil crack depth is quite different from the final crack depth under dry-wet cycle of various climate conditions, but further studies about the influence mechanism of wet-dry cycles on crack developing depth and intensity degeneration, and so forth are not carried out, nor is the final crack depth calculation model that reflects the dry-wet cycle process established. There are some differences between the proposed initial crack developing depth model and the actual project, and as a result, the model cannot be used to guide the practice. Based on this, it is mainly the soil body's initial state that is analyzed in this paper, the cumulative damage model of expansive soil after repeated of dry-wet cycles is established, the time rule for water content changes is introduced, the calculation model for the expansive soil crack developing depth after dry-wet cycle is established and its applicability is analyzed and verified. The establishment of this calculation model provides theoretical basis for guaranteeing the safety of expansive soil slope project and calculating depth of processing.

## 2. Crack Development Mechanism and Its Influencing Factors

### 2.1. Crack Development Mechanism

As an anisotropic nonideal rigid-plastic material, the expansive soil allows the soil body to produce a certain amount of deformation without damage; this is mainly because of its own cohesion. That is to say, crack will not appear immediately after being pulled and the soil body will crack only when the tensile stress increases to the amount of deformation and exceeds the ultimate tensile strain that causes cracks.

**Figure 1:**
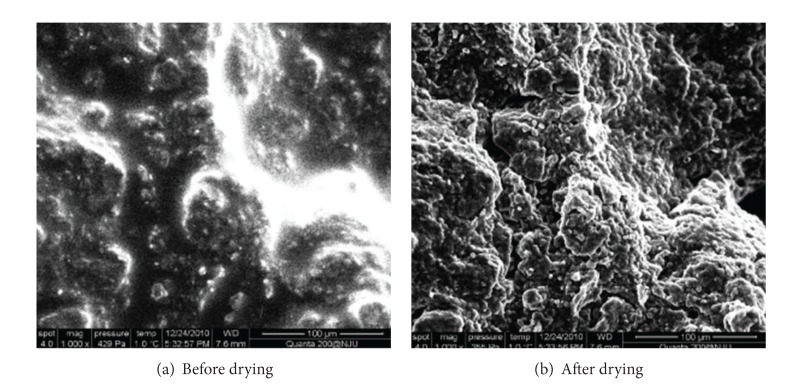
ESEM images of Romainville expansive soil during drying.

Supposing the expansive soil is saturated initially, the vapor pressure of the air above the surface of the soil body is balanced with the pressure inside the soil body, and there are not any energy exchanges between the soil body and the atmosphere. With changes of the external environment, such as with temperature increase and thermal radiation enhancement, temperature of the surface soil particles will increase after being heated, kinetic energy of the free water between particles will increase with increasing temperature and the water molecules become very active and gradually get rid of bondage, transforming into water vapor to escape into the atmosphere, which makes the air humidity around the surface soil increase, breaking the original balance. As a result, the surface soil moisture begins to decrease, and free water of the deep soil gradually goes upward to make a supplement, causing deep soil moisture and surface soil moisture to change. Since water loss of the surface soil is faster than that of the deep soil, thus forming the moisture gradient from surface to the deep, resulting in increased matrix suction inside the soil. The air begins to enter the soil body when the matrix suction is greater than the air suction value of the soil, then the expansive soil gradually gets nonsaturated. Free water is more likely to become water vapor and evaporate under the roles of matrix suction, while capillary water around the small pore of soil particles is gradually released to become free water. The lost capillary water will be complemented by the penetration-adsorption bound water of the diffusion layer and the directional-adsorption bound water, the soil particles will have a closer connection with each other although they have lost their free water and the bound water film has become thinner, volume shrinkage, and deformation after losing water is displayed macroscopically. The microstructure change of the expansive soil before and after drying is as shown in Figure 1 [5]. It shows that when the water content is high (Figure 1(a)), the soil pores are filled with water, the soil particle boundary is blur, and the hydration phenomenon is obvious; when it is dried due to water loss (Figure 1(b)), the water film disappears, the soil particles and pores are clear and visible, but the scale decreases when compared with before, and microscopic cracks also appear in local area.

The expansive soil is a material of homogeneity that is formed by the mixture of clay minerals such as the montmorillonite, the illite, and other organic materials. Neither the original state nor the laboratory sample preparation could guarantee their homogeneity, and there must be some natural defects in the internal structure of the soil body. With decrease of free water in the soil, the bound water film around the soil particles becomes thinner, the pores become bigger, and further more the matrix suction increases, when it reaches a certain limit value, namely, the maximum tension strain that the soil can withstand, cracks will appear in where the natural defects exist.

Transpiration that is caused by sunlight is a key external reason for water loss of the expansive soil. The transpiration intensity affects the intensity of water content changes inside the soil body, namely, water content gradient, thus affecting the matrix suction, which causes soil deformation together with gravity. The more obvious the transpiration is, the greater the moisture gradient and the matrix suction will be, the greater the local deformation will be, and it will be more easily for the cracks to develop. The more obvious the water loss after crack development is, the greater the crack depth will be. It is to say that the soil cracking is mainly because of the tensile force that is caused by different water loss rate in soils of different depths, the tensile force leads to uneven shrinkage and deformation inside the soil, the crack will appear in the weakest place when that deformation reaches a certain value.

### 2.2. The Crack Development's Influencing Factors

Expansive soil is composed of soil particles, water, and gas that exist in pores between the soil particles. The pores between soil particles can be seen as a kind of microfissures, and the visible macroscopic cracks are caused by cracking of these microfissures on load. The essence of expansive soil cracking is the external manifestation of uneven volume shrinkage and deformation. The stronger the volume's uneven shrinkage is, the higher the development degree of crack will be. While the soil shrinkage is closely related to its own initial state, the external environmental state and so on.

(*1) The Original State of the Expansive Soil.* The soil's initial states affecting expansive soil's cracks development factors include the montmorillonite content, the moisture content, the structural morphology, the compaction degree, Poisson's ratio, and the content of organic salt. Compared with other clay minerals, the montmorillonite has stronger hydrophilic characteristic, can form thicker hydrated film, and can provide greater shrinkage space during the drying and dehydration process. Therefore, the higher the montmorillonite content is in the expansive soil, the easier the crack development is and the greater the development width is. Under conditions of high water content, the clay particles are coated with a thick hydrated film, the space between particles is larger; in the process of dehydration and drying, the free water is gradually evaporated, the space between particles becomes smaller, meanwhile air goes into the soil constantly, the bound water in the hydrated film is gradually released to become free water and as a result the film becomes thinner, under the roles of suction and lubrication of free water, the soil particles are rearranged and gradually move closer to each other, pores become smaller gradually, which will be exhibited as volume shrinkage deformation macroscopically, and cracks will appear when the tensile stress of a weak zone reaches the ultimate tensile strength. Seeing from the soil's microstructure morphology, laminar-flow structure is conducive to the generation of expansive soil cracks, has good pores connectivity, and is helpful for moisture migration and cracks cut-through after expansion. Poisson's ratio reflects the relationship between vertical and horizontal deformations. A high Poisson ratio of soil means that the soil is influenced more by vertical deformation than by lateral deformation, it is softer, the contact lubrication between the soil particles is better, the mobility is better, and it is easier for the particles to slip and dislocate; therefore, volumetric strain is not prone to occur in the soil body [13]. Under high degree of compaction, the expansive soil's structure is dense, the connection between the particles is compact, the injury degree of the original structure is small, the volume content of free water is low, the infiltration ability is not good, and the matrix suction is strong when the surface soil loses water loss and shrink; therefore, it is not easy for the soil body to crack. The expansive soil contains a relatively large amount of soluble organic salt, which is dissolved when meeting water, and then number of pores inside the expansive soil structure increase, the infiltration coefficient becomes larger, which is helpful for soil evaporation and diffusion of water vapor; as a result, the soil is prone to crack.

(*2) Influences of External Environment.* The external environmental factors affecting expansive soil crack development include the evaporation rate, the ambient temperature, the relative humidity, and the wind speed [14]. Macroscopically, water evaporation means the process of free water transforming from a liquid state to a gaseous state; microscopically, water evaporation is actually the process of water molecules overcoming the intermolecular forces within the liquid with the help of kinetic energy, and then escaping from the liquid surface to the atmosphere. For the soil, its evaporation process is much more complex than pure water, because it is affected by the ambient temperature, the relative humidity, the air flow rate, the solar radiation, and many other external factors [15, 16]. Hillel [17] pointed out that the occurrence and maintenance of water evaporation in soil must meet three conditions: (1) there is a continuous supply of calories on the soil surface; (2) the atmospheric vapor pressure is lower than the vapor pressure on soil surface; (3) there is a continuous water supply from the internal soil to the evaporation surface (soil/air interface).

**Figure 2:**
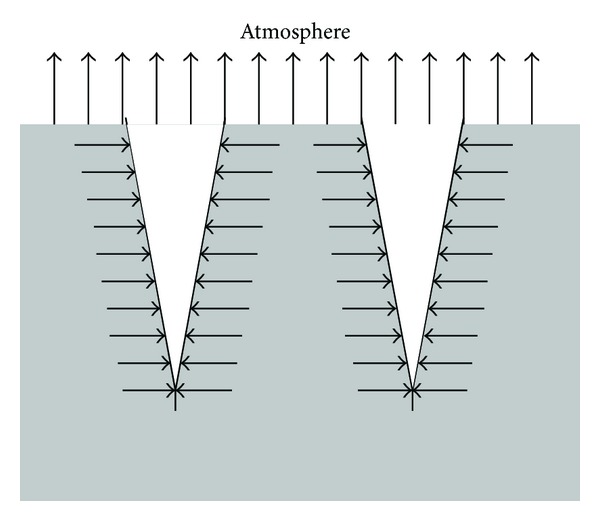
Moisture evaporation model of soil with fissures.

**Figure 3:**
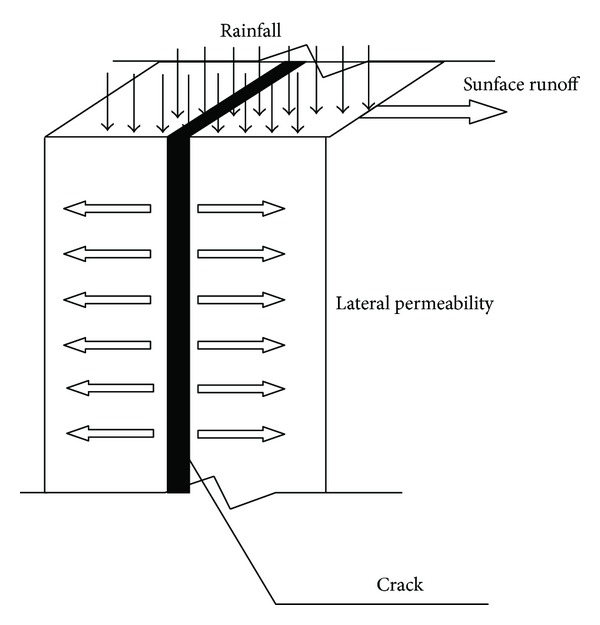
Rainfall infiltration model of soil with fissures.

The ambient temperature of expansive soil is relatively high, the relative humidity on soil surface is low, and the wind speed is higher, thus resulting in a higher velocity and kinetic energy of water molecules. The adsorption bound water of clay particles transits to free water and the thickness of diffusion layer becomes thinner; meanwhile, the viscosity of water, the surface tension, and the water holding capacity of the soil decrease and the ability of clay particles to bound water molecules is reduced and so does the resistance that the water molecules need to overcome in order to escape into the atmosphere. Wind makes the water molecules that has escaped onto the soil surface drift rapidly, the relative humidity gradient between soil - air interface further increases, and water loss and the corresponding evaporation rate increase at the same time, thus leading to the increase of suction development rate in the surface soil. The resulting surface tensile stress field will exceeds tensile strength of the soil in a relatively short period of time, thus cracks will appear. The evaporation and rainfall infiltration of soil with cracks is as shown in Figures 2 and 3.

## 3. The Initial Crack Depth Considering Water Content Change Rate

The above-mentioned crack development mechanism and its influencing factors analysis indicate that the decrease of water content makes the saturated expansive soil lose water to become nonsaturated, the matrix suction inside the soil body increases at the same time, and then the soil shrinks and deforms due to water loss, which is accompanied by growth of the internal tensile stress, resulting in growth of the soil's strain. That is to say, the deformation of the soil is because of the strain *ε*_*w*_ caused by tensile stress, which is caused by the increase of matrix suction after water loss of the soil, and fissures in the soil begins to crack when the strain reaches the limit deformation *ε*_*w*_ > *ε*_max⁡_.

In order to determine the laws of water content changes with time in the process of drying, the water loss rate is defined to be soil samples of unit height, water content changes in unit time is

(1) ΔW=WSW0×Δt×100%.

In the formula, *W*_*S*_ is the water loss per unit time, *W*_0_ is the soil's original volumetric water content, and Δ*t* is the elapsed time of dehydration process.

The water loss rate is related with such factors like time, ambient temperature, relative humidity, initial moisture content, and the basic nature of the soil. Changes of the water loss rate will lead to the generation of the soil's strain *ε*_*W*_ = *α*_*i*_Δ*W*, where, *α*_*i*_(*x*, *y*, *z*) is the expansion-contraction coefficient, representing the soil's strain increment when the unit water content increases or decreases, and it is related to the soil's inherent physical nature and reflects the soil's strain intensity with changes of water loss rate, namely, the expansion-contraction deformation capability. Under normal conditions, the *α*_*i*_ in the moisture absorption process is different with the *α*_*i*_ in the dehumidification process, it is also different in different wet-dry cycles, and its general law may be obtained through related tests. As a expansion-contraction coefficient, *α*_*i*_ does not change with the change of direction for a certain point of the ideal isotropic body; that is to say, it can be regarded as linear-expansion coefficient *α*. Therefore, strain that is caused by dehydration shrinkage can be rewritten as.

(2) εw=αΔW.

Suppose the soil particle is incompressible, according to the principle of effective stress, the stress state of unsaturated soils can be expressed as *σ* − *u*_*a*_ and (*u*_*a*_ − *u*_*w*_), where *u*_*a*_ is the pore air pressure, (*u*_*a*_ − *u*_*w*_) is the matrix suction, and *σ* − *u*_*a*_ is the net normal stress. Fredlund thinks that for the isotropic and linear elasticity unsaturated soil, the soil principal strain [15], can be obtained according to the elastic theory, considering the convergence with saturated soil:

(3) εx=(σx−ua)E−μE(σz+σy−2ua)+(ua−uw)H,εy=(σy−ua)E−μE(σx+σz−2ua)+(ua−uw)H,εz=(σz−ua)E−μE(σx+σy−2ua)+(ua−uw)H.

In the formula, *H* is an elastic constant associated with (*u*_*a*_ − *u*_*w*_). When the unsaturated soil is under the static condition of *K*_0_, the strain occurs in a fixed direction; that means that *σ*_*x*_ = *σ*_*y*_ = 0, *ε*_*x*_ = *ε*_*y*_, before the cracks appears on the horizontal surface. When they are put into (3), it is seen that

(4) (σx−ua)=μ(1−μ)(σz−ua)−E(1−μ)H(ua−uw).

With the scope of crack depth under the state of stress balance, both (*σ*_*x*_ − *u*_*a*_) and (*σ*_*y*_ − *u*_*a*_) are zero; for example there is only vertical stress in cracks. Considering the water loss of the soil may cause soil strain, (2) is put into (4) to obtain the soil's limit strain capacity when cracking, namely, the strain capacity that is generated under the joint roles of matrix suction and effective stress after losing water:

(5) εw=αΔW=3(1−2ua)E(σz−ua)+3H(ua−uw).

For isotropic body, when one-dimensional strain occurs in the soil, *σ*_*x*_ = *σ*_*y*_ ≠ *σ*_*z*_, *ε*_*x*_ = *ε*_*y*_ and the volumetric strain is

(6) εv=σz−uaE−γ(2σx−2ua)E+ua−uwH.

When put (5) into (6), it can be obtained that

(7) $$\begin{array}{c} \varepsilon_{v}=\frac{1-\mu -2\mu^{2}}{E\left(1-\mu \right)}\left(\sigma_{z}-u_{a}\right)+\frac{1+\mu }{\left(1-\mu \right)H}\left(u_{a}-u_{w}\right). \end{array}$$

**Figure 4:**
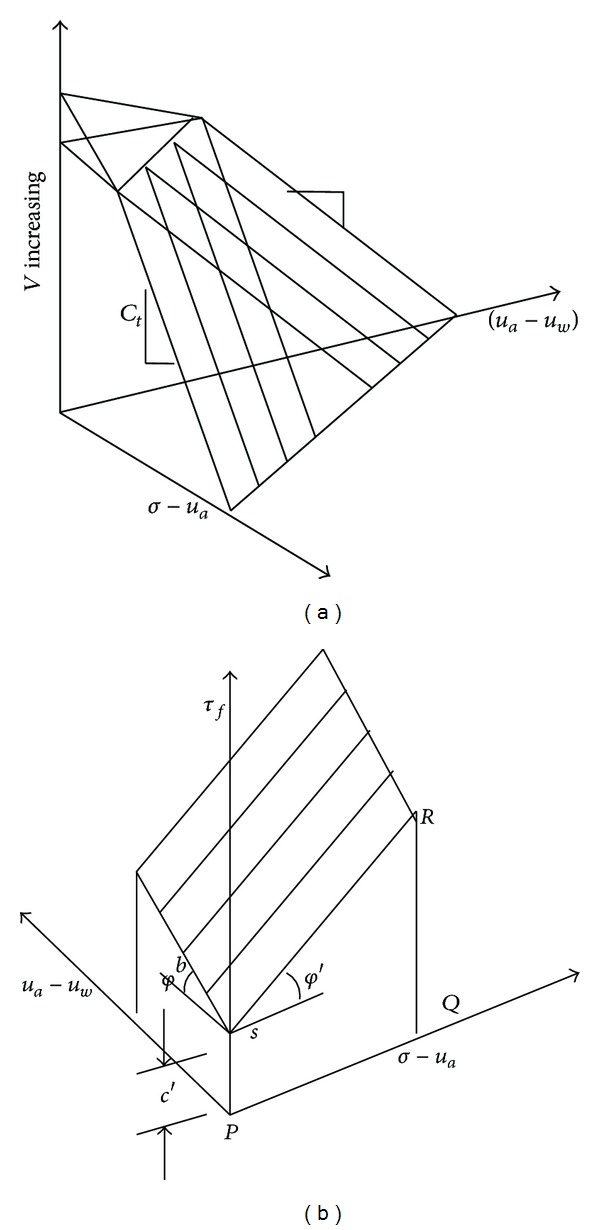
The compression state model of unsaturated soil (a) and its shear strength (b) [10].

Let *c*_*t*_ = 1 − *μ* − 2*μ* ^2^/*E*(1 − *μ*), *c*_*a*_ = 1 + *μ*/[(1 − *μ*)*H*], where *μ* is Poisson's ratio of the soil, *c*_*t*_ is the volumetric strain which is generated by the increase of net unit stress, and *c*_*a*_ is the volumetric strain which is generated by the increase of unit matrix suction. The concrete meaning of the parameters may be showed by the unsaturated soil's state of strain when it is compressed (as shown in Figure 4(a)). It can also be seen from the figure that either under the roles of horizontal net stress, matrix suction, or their joint roles, the volumetric strain will grow.

Cracks continue to develop from the tip when the horizontal tensile stress exceeds the soil's tensile strength, the horizontal stress also reduces accordingly, and finally cracks stop developing when a balance between the horizontal stress, the matrix suction, and the effective stress is achieved. Suppose the saturation of the crack tip remains unchanged when the crack is developing, thus the strain that is caused by the change of external soil strain will be the same with the strain that is caused by the change of matrix suction. Therefore, it can be assumed that *c*_*t*_ = *c*_*a*_ and as a result, *E*/*H* = (1 − 2*u*) is established for the isotropic earth body under the conditions of a one-dimensional strain; that is, the state of stress at the crack tip has nothing to do with the *E*/*H* ratio [10].

According to experimental research and field investigation, the crack is generally not deep in the initial stage of surface cracking, and it continues to develop in depth and width with further changes in water content. Therefore, if the pore air pressure *u*_*a*_ is zero at the beginning of crack developing, then

(8) σz−ua=γZ,

where *r* is the soil unit weight and *z* is soil depth.

Put formula (8) into (5) an expression of matrix suction at the depth of *Z* will be obtained:

(9) (ua−uw)=EαΔW−3γΖ3(1−2μ).

Put formula (9) into (5), to obtain formula of the crack's tensile stress

(10) (σx−ua)=3γΖ−EαΔW3(1−μ).

**Figure 5:**
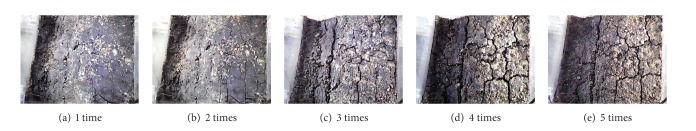
The expansive soil cracks of different dry-wet cycles times.

For unsaturated expansive soil, the soil's tensile strength *t* [8] can be expressed as:

(11) t=−μ(1−μ)[c′+(ua−uw)tanφb]cot⁡φ′,

where *φ*′ describes how the strength increases with total pressure, *φ* ^*b*^ describes how the strength increases with matrix suction (as shown in Figure 5(b)), and (*u*_*a*_ − *u*_*w*_) is the matrix suction and *c*′ is the effective cohesion.

Matrix suction is generated inside the expansive soil due to water loss, and net horizontal stress is produced on the soil surface. When this stress increases until it makes the soil deformation reach the maximum, cracks will appear and develop; therefore, the crack's tensile stress value at the beginning is *t* = (*σ*_*x*_ − *u*_*a*_); that means that formula (10) and formula (11) are equivalent, when put (9) into (11),

(12) 3γΖ−EαΔW3(1−μ)  =−μ(1−μ)[c′+(EαΔW−3γΖ3(1−2μ))tanϕb]cot⁡ϕ′.

After finishing, the initial crack depth formula of unsaturated expansive soil when considering water loss rate is obtained:

(13) Z=NEαΔW+Mγc′N,

where *M* = *μ*(2*μ* − 1) and *N* = (1 − 2*μ*)−(*μ* − 1)tan*φ* ^*b*^.

It can be seen clearly from this formula that, in addition to the properties of the soil itself such as Poisson's ratio, elastic modulus, linear expansion coefficient, soil bulk density, and effective cohesion, the initial crack developing depth is also related to the increase rate of shear strength that is caused by matrix suction and the water loss that is caused by external environment. That is to say that the initial crack depth deepens the increase of linear expansion coefficient, elastic modulus, water loss rate, and shear strength's increase, and it shallows with the increase of Poisson's ratio, bulk density, and cohesion, which is the same as the analysis results of factors influencing cracks development and the formula can correctly reflect the process of expansive soil transforming from the saturated state to the unsaturated that is caused by water loss and shrink, and the crack depth changes that constantly develops until stop.

## 4. Crack Developing Depth Formula of Cumulative Damages

### 4.1. The Cumulative Damage Analysis of Dry-Wet Cycles on the Soil Body

As a high plasticity clay with fractured characteristic and swell-shrink characteristic, the expansive soil is extremely sensitive to climate change, it often undergoes expansion-contraction deformation due to repeated wetting-drying cycles, resulting in the accumulation of deformation, its strength continue to decay and eventually the accumulation of soil deformation, and strength injury lead to the occurrence of the expansive soil slope's project disasters. When the saturated expansive soil loses water, the soil moisture content decreases gradually and the matrix suction increases, and thus the soil becomes nonsaturated and the elementary tension crack appears. As the drying process continues, when the tensile stress at the bottom of the elementary crack is less than the tensile stress at a certain part of the surface, the secondary crack appears. After rainfall, because of the presence of cracks, rainwater quickly goes into the soil along the cracks, the penetration speed is increased, and the soil becomes saturated after sucking water, as a result the crack is closed again due to expansion. Repeated wetting-drying cycles damage the soil and what is displayed macroscopically is the differences of the crack surface morphology in different wet-dry cycle times which is as shown in Figure 5. By analyzing the crack developing process under dry-wet cycles in actual slope project, Li [18] think that when the tensile stress that is caused by the soil water loss reaches a certain level the tension cracks will appear at the weak position of the soil. Although the cracks will be closed after meeting water, the internal damages cannot fully recover, cracks appear in the soil mass after dried, and then after sucking water it becomes saturated; but the original cracking position cannot be completely closed due to damages. Therefore, cracks will firstly appear in the original cracking position when loosing water again. Every time the soil cracks, damages will be done to the soil body, supposing that every dry-wet cycle will cause the same tensile stress inside the soil body, and then the cracks will gradually deepen or new secondary cracks will appear.

The dry-wet cycle of expansive soil is actually because of repeated matrix suction's loading and unloading that is caused by moisture changes, which leads to irreversible accumulation damages to the internal structure of the soil, the effects produced by dry-wet cycles are closely related with the cycle process control parameters and is similar to the cyclic loading process of solid mechanics. The stress levels can be considered to be different change rates of water content caused by different dry-wet cycle ranges, namely, the water loss rate. The irreversible damages to the soil structure causes the decrease of the soil's overall tensile strength and shear strength, as a result, even the same magnitude of dry-wet cycles will also play a catalytic role in the development of cracks. And of course, crack depth will not extend unlimitedly, with the increase of the number of wet and dry cycles, damages continue to accumulate, the soil becomes looser, the major crack deepens, microcracks gradually increase, and finally, the effects of each dry-wet cycle will be absorbed by the microcracks, thus the expansive soil's elementary crack depth will not increase any more; that means that it has reaches a final depth of expansive soil cracking.

### 4.2. The Expansive Soil Crack Depth Calculation under Cumulative Damages

The research results of Lü et al. [19], Zheng [20] show that with the increase in the number of dry-wet cycles, the decrement of strength and deformation shows a decreasing trend. That is, each dry-wet cycle causes different damages, which shows a law of nonlinear diminishing. Therefore, supposing the stress level of the *i*th time is *S*_*i*_ (referring to the dry-wet cycle here), the caused damage fraction [21, 22] is as follows: *D*_*i*_ = 1/*N*_*i*_, in the formula and *N*_*i*_ represents the number of fatigue damage under the current stress level of *S*_*i*_. What should be explained here is that, although *S*_*i*_ is used to represent the stress level (i.e., wet and dry cycle) of each time, the value of *i* varies every time and the ranges of each dry-wet cycle are not necessarily the same; therefore, the soil damage fraction *D*_*i*_ is also different.

The cumulative damage under multilevel cyclic stress level is:

(14) D=∑iDi=∑niNfi,

where *D*_*i*_ is damage fraction under the roles of the stress level *S*_*i*_, *n*_*i*_ is the sample's actual cycle times under the roles of the stress level *S*_*i*_; and *N*_*fi*_ is the number of cycles when the sample reaches a steady residual strength under the stress level *S*_*i*_.

In order to accurately reflect the damage of each dry-wet cycle to the soil and the impacts of their cumulative damages on the crack development, the expansive soil is supposed to be an isotropic body and various basic physical quantities of the soil affecting cracks development have the same damage laws, namely, under the roles of the same stress level *S*_*i*_ and various physical quantities have the same nonlinear damage ratios, that is, after the *i*th dry-wet cycle, the various physical quantities reckoning in damages are

(15) Ei=E(1−∑iDi),  ci′=c′(1−∑iDi),γi=γ(1−∑iDi),  μi=μ(1−∑iDi).  

Then the calculation formula for crack developing depth under the stress level *S*_*i*_ is

(16) Zi=NEiαΔWi+Mγici′N.

Put (15) into (16), the calculation formula for the final crack developing depth considering cumulative damage will be obtained:

(17) Z=∑iZi=(1−∑iDi)[μ(2μ−1)γc′A+EAα∑iΔWi],

where *A* = [1 − *μ*(1 − ∑_*i*_ *D*_*i*_)(2 + tan*φ* ^*b*^)].

All the intrinsic basic property indicators of the expansive soil used in the formula, such as *μ*, *γ*,  *c*′, *α*, *E*, and *φ*, can be determined by laboratory tests. Because the assumed precondition of the formula is that various physical quantities have the same nonlinear damage ratio, the accumulated damage amount ∑_*i*_ *D*_*i*_ that is caused by wet and dry cycle can be replaced by the ratio of expansive soil residual strength and peak intensity obtained through different dry-wet cycle tests.

## 5. Discussions

In the state of evaporation and dryness, the expansive soil deforms due to swelling, which is accompanied by the initiation and development of cracks. Cracks appearing after rainfall will be filled with water quickly and become saturated, and meanwhile the water seeps all around. The presence of cracks will significantly enhance the infiltration capacity, the soil's saturation speed is faster, but the cracks cannot be fully recover due to damage effect and can only be substantially closed. Under the roles of reevaporation, the original cracks communicate with the atmosphere well because they were not fully closed, and dehydration speed is faster, thus prompting the crack development. The continuation of evaporation and the increase of the soil's matrix suction prompt the cracks to develop in depth, or prompt the appearance of new cracks when the deformation exceeds the limiting value.

The sustained effect of dry-wet cycles is accompanied with the initiation and development of expansive soil cracks. The above analysis also shows that the formation process of cracks is a nonlinear process, which is difficult to describe in the actual simulation analysis. All the existing crack development depth calculation models [12, 20] are derived from analysis of the soil's inherent physical properties, which starts from the linear elastic theory. Li et al. analyzed the factors affecting expansive soil crack depth think that the crack depth of unsaturated expansive soil tends to decrease with the increase of effective soil cohesion and internal friction angle, and the effective cohesion has a relatively stronger influence on the crack depth [13]. The changes of the material properties with dry-wet cycle process and time are considered in neither of these calculation models, resulting in the differences between the simulation results and the real situation. In fact, the crack development depth is the result of many factors; in addition to the original state of cracks, the influences of external condition changes on the crack development cannot be ignored.

Starting with the study of crack development mechanism, the internal and external factors affecting the expansive soil crack development are analyzed in detail in this paper, the change rate of water content inside the soil is considered to play a key role in the expansive soil crack development depth, and the initial crack depth is deduced accordingly. Different dry-wet cycle will damage the soil body differently, the gradually accumulated irreversible damages weaken the soil strength and its inherent nature, and thus the crack development is gradually deepened. Supposing the damage ratio of the soil's inherent nature are the same to each other, and the computational formula for the final development depth of expansive soil cracks is deduced based on this. Judging from the formula, the expansive soil crack gradually deepens to a certain extent under the precondition of cumulative damages until it stops developing, and in addition to the soil's inherent nature parameters, the crack depth is also relative with and deepens with the accumulations of damages and water loss rate. The cumulative process of the expansive soil crack depth development can be better reflected.

## 6. Conclusions

By analyzing the developing mechanism and influencing factors of expansive soil cracks, the formulas for the initial and final crack developing depths are deduced and the flowing conclusions are made.

1. The expansive soil allows a certain amount of deformation but without damages and cracks because of its own cohesion, transpiration such as sunlight will accelerate the water loss of thus the water content gradient is formed inside the soil body, the matrix suction increases, and the greater the local deformation is, the deeper the cracks will be, namely, the mechanism of expansive soil crack development is that, the tensile force generated in different depth because of different water loss rate leads to uneven internal contraction deformation of the soil, and then tensile cracks will appear in the weakest position when the deformation reaches a certain value.
2. By analyzing the factors affecting the unsaturated expansive soil crack development depth, it is thought that with the increase of montmorillonite content, organic salt content, compaction degree, as well as the decrease of Poisson ratio, the expansive soil cracks become deeper, and, with the increase of evaporation rate, environmental temperature, wind speed, the relative humidity, the expansive soil cracking is quicken.
3. Starting from the basic theory of linear elastic mechanics, the key factors influencing the crack developing depth of expansive soil, water loss rate; is introduced, and the calculation model for the unsaturated expansive soil developing depth is established. The model can correctly reflect the process of the expansive soil changing from the saturated to the unsaturated state due to water loss and shrinkage, and the initial crack depth that develops continuously until eventually it stops.
4. The changes of expansive soil water content under the roles of dry-wet cycles are analyzed, and it is thought that each dry-wet cycle will lead to irreversible cumulative damage to the soil's internal structure, the soil becomes looser, the main crack gradually deepens and the number of microfractures increases gradually. In the end, the effects of each dry-wet cycle are absorbed by the formed microcracks, the depth of the expansive soil's main crack will no longer increase because it has reached the final depth of expansive soil cracks. Based on the cumulative damage analysis of dry-wet cycle to the expansive soil, the final depth of the expansive soil is established, and parameter choices in the formula are analyzed and discussed.

Starting from the expansive soil water content changes caused by dry-wet cycles, the concept of water loss rate is introduced in this paper, and the initial and final crack depth values in theory are obtained. But the dry-wet cycle of the expansive soil is an extremely complex process, and the change rule of the soil's basic physical properties has not been mastered by people, meanwhile in the formula of crack depth, statistical analysis of the cumulative damages is only done on the basis of hypothesis and simplification, and the study about the impact of other external environment changes on the water loss rate and the cumulative damage research is not thorough, therefore in calculation, it is necessary to determine parameters in the obtained formula by dry-wet cycle tests. Study the soil damage process caused by dry-wet cycle systematically, explore the damage law of various physical quantities, analyze sensitivity to the influence of crack depth thus to simplify the calculation formula of crack depth, these problems will be the research emphasis of the author in the future.
